# Renal cell carcinoma: a review of biology and pathophysiology

**DOI:** 10.12688/f1000research.13179.1

**Published:** 2018-03-12

**Authors:** Shahzaib Nabi, Elizabeth R. Kessler, Brandon Bernard, Thomas W. Flaig, Elaine T. Lam

**Affiliations:** 1 Division of Medical Oncology, Anschutz Medical Campus, University of Colorado Denver, Aurora, CO, USA

**Keywords:** renal cell carcinoma, biology, von Hippel Lindau, VHL, PBRM-1, BAP-1, HIF, mTOR, Glutaminase, Immunotherapy

## Abstract

Over the past decade, our understanding of the biology and pathophysiology of renal cell carcinoma (RCC) has improved significantly. Insight into the disease process has helped us in developing newer therapeutic approaches toward RCC. In this article, we review the various genetic and immune-related mechanisms involved in the pathogenesis and development of this cancer and how that knowledge is being used to develop therapeutic targeted drugs for the treatment of RCC. The main emphasis of this review article is on the most common genetic alterations found in clear cell RCC and how various drugs are currently targeting such pathways. This article also looks at the role of the immune system in allowing the growth of RCC and how the immune system can be manipulated to reactivate cytotoxic immunity against RCC.

## Introduction

Renal cell carcinoma (RCC) is a heterogeneous group of cancers arising from renal tubular epithelial cells that encompasses 85% of all primary renal neoplasms ^1, 2^. The most common subtypes of RCC are clear cell RCC (ccRCC), papillary RCC, and chromophobe RCC ^1^. The remaining 15% of tumors of the kidney consist of transitional cell carcinoma (8%), nephroblastoma or Wilms’ tumor (5–6%), collecting duct tumors (<1%), renal sarcomas (<1%), and renal medullary carcinomas (<1%). The incidence of RCC varies widely in different parts of the world, and the highest incidences are in North America and the Czech Republic ^2^. In the US, there are 64,000 new cases of RCC and 14,000 RCC-related deaths each year ^3^. Age, race, and gender also play a role in this disease. RCC is more common in males above the age of 60 (median age for RCC is 65), and the highest incidence is in the sixth to eighth decades of life ^4^. Within the US, Caucasians, African-Americans, Hispanics, and Native Americans have a higher incidence of RCC as compared with Asian-Americans or Pacific Islanders ^5, 6^.

Multiple risk factors for RCC along with their pathophysiologic mechanisms have been described. These include both genetic and acquired risk factors. The two most common genes involved in the pathogenesis of RCC are the Von Hippel–Lindau ( *VHL*) gene and the protein polybromo-1 ( *PBRM-1*) gene. These will be discussed separately in this article. The most common acquired risk factors for RCC are smoking, hypertension, obesity, chronic analgesic use, and diabetes ^7^.

## Genetic alterations in renal cell carcinoma

The most common genetic alteration associated with the development of ccRCC is loss of the short arm of chromosome 3 (loss of 3p). This alteration is seen in approximately 95% of cases of ccRCC. The most common genes involved in the pathogenesis of ccRCC include *VHL*, *PBRM-1*, *SETD2*, *BAP-1*, *KDM5C*, and *MTOR* ^1, 8^. Other genetic alterations include gain of 5q (69%), partial loss of 14q (42%), 7q gain (20%), 8p deletion (32%), and 9p loss (29%) ^8^.

### Von Hippel–Lindau gene

*VHL* is a tumor suppressor gene that plays a pivotal role in the development of ccRCC. *VHL* can be altered and transmitted in an autosomal dominant fashion (VHL disease) or in a sporadic manner. Although inherited VHL disease is rare, understanding the molecular basis of VHL disease and the identification of the VHL suppressor gene have provided great insight into the pathogenesis of sporadic disease. It is estimated that 50–60% of patients with sporadic ccRCC have an abnormality of the *VHL* gene ^9– 11^. Other, more sophisticated studies have suggested that *VHL* gene alterations through genetic and epigenetic mechanisms can be found in up to 90% of ccRCC cases ^12^.

A “two-hit” hypothesis has been described and validated in patients with VHL disease-related development of RCC (and other tumors). Based on this hypothesis, individuals with VHL disease are born with one inactivated copy of the *VHL* gene in all cells while the other copy of the gene is normal.For tumorigenesis to take place, there must be a loss of function of the second gene copy as well. This “second hit” usually takes place as a result of somatic mutation or deletion of the allele. In patients with sporadic RCC, inactivation of both VHL alleles usually takes place via somatic mutations.

The product of the *VHL* gene is a protein called pVHL, which acts as a tumor suppressor protein. VHL protein forms complexes with several other proteins in the cell, including elongin B, elongin C, and cellulin 2. The resulting complex (called the VBC complex) helps in the proteasomal degradation of several intracellular proteins. One of the major functions of the VHL gene product is regulating the levels of several intracellular proteins, including hypoxia-inducible factor 1 alpha and 2 alpha (HIF1A and HIF2A) ^13, 14^. These intracellular proteins, when bound with each other, serve as transcription factors by binding to the DNA, resulting in upregulation of messenger RNA (mRNA) that codes for several growth factors, including vascular endothelial growth factor (VEGF), platelet-derived growth factor beta (PDGFB), and transforming growth factor alpha (TGFA). These growth factors play a vital role in the development of highly vascular tumors (such as ccRCC) associated with *VHL* gene alterations. The mRNA also codes for other proteins and enzymes responsible for controlling proteins in the extracellular matrix.

**Figure 1.**
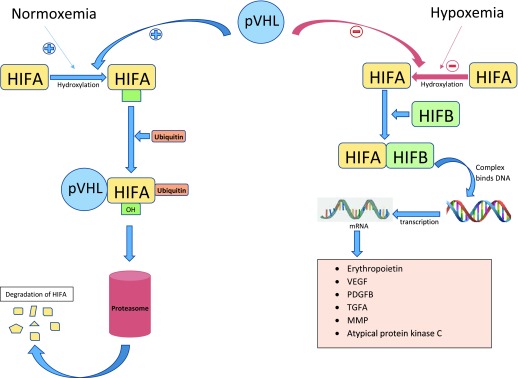
VHL/HIF axis. Under normal oxygen tension, HIF1A and HIF2A are hydroxylated on proline residues and bind the pVHL, resulting in the polyubiquitination of HIFA, which targets it for proteasomal degradation. Under conditions of hypoxemia or in the absence of pVHL, hydroxylation of HIF1A and HIF2A does not occur and HIFA accumulates and dimerizes with HIFB and acts as a transcription factor, resulting in increased mRNA levels coding for VEGF, PDGFB, TGFA, erythropoietin, and extracellular matrix protein. HIFA, hypoxia-inducible factor alpha; HIFB, hypoxia-inducible factor beta; MMP, matrix metalloproteinase protein; mRNA, messenger RNA; PDGFB, platelet-derived growth factor beta; pVHL, protein of Von Hippel–Lindau gene; TGFA, transforming growth factor alpha; VEGF, vascular endothelial growth factor.

Under normal oxygen tension, HIF1A and HIF2A are hydroxylated on proline residues and bind the pVHL, resulting in polyubiquitination of HIFA, which targets it for proteasomal degradation ( Figure 1) ^15, 16^. Under conditions of hypoxemia or in the absence of pVHL, hydroxylation of HIF1A and HIF2A does not occur and HIFA accumulates in the cell and dimerizes with hypoxia-inducible factor beta (HIFB). The HIFA–HIFB complex then migrates to the nucleus and acts as a transcription factor, resulting in increased mRNA levels coding for VEGF, PDGFB, TGFA, erythropoietin, and extracellular matrix proteins ^14, 17^.

From a therapeutic standpoint, inhibitors of VEGF are typically used as first-line therapy for the treatment of metastatic ccRCC. Sunitinib and pazopanib are two commonly used tyrosine kinase inhibitors that target and block vascular epidermal growth factor receptor (VEGFR) ^18, 19^. Axitinib, cabozantinib, lenvatinib, and sorafenib are other tyrosine kinase inhibitors that block VEGFR. Bevacizumab, a monoclonal antibody that directly targets VEGF, is also a treatment option for ccRCC. In addition, drugs that block HIFA would theoretically block this pathway, resulting in a decrease in the production of angiogenic factors (such as VEGF and PDGFB) and a decrease in tumor growth. Selective HIF2 antagonists PT2399, PT2385, and PT2977 are under investigation ^20– 22^. PT2399 has been shown to cause regression in preclinical models (cell line and tumorgraft/patient-derived xenograft) of pVHL-defective ccRCC ^20, 21^. PT2385 has been evaluated as monotherapy in a phase 1 study in patients with metastatic ccRCC and has been shown to have a favorable safety profile and early evidence of efficacy ^22^. PT2977, a more potent HIF2a antagonist, is being investigated in phase 1 clinical trials for the treatment of solid tumors and ccRCC and in a phase 2 clinical trial for the treatment of patients with VHL disease.

### Protein polybromo-1 gene

*PBRM-1* is also a tumor suppressor gene that plays an important role in the pathogenesis of ccRCC. It encodes a protein called BAF180, which is a subunit of the nucleosome remodeling complex. The nucleosome remodeling complex is a complex group of proteins that control the expression of certain genes by accessing the condensed part of the DNA. It is unclear how BAF180 acts as a tumor suppressor protein; however, several animal studies have shown that it plays a role in controlling the cell cycle and replicative senescence. Re-introduction of *PBRM-1* in *PBRM-1*-deficient cell lines typically produces cell cycle arrest. Thus, a mutated *PBRM-1* gene would result in an abnormal/malfunctioning BAF180, which would result in unchecked cell growth and subsequent tumorigenesis ^23, 24^.

### BRCA1-associated protein-1

BRCA1-associated protein-1 ( *BAP-1*) is a tumor suppressor gene located on 3p. This gene is mutated in approximately 15% of ccRCC cases. *BAP-1*-mutated tumors tend to be more aggressive and are generally related to a worse prognosis ^25^. Like other tumor suppressor genes, *BAP-1* plays a role in the suppression of cell proliferation. It does so by interacting with a transcription protein called host cell factor-1 (HCF-1). HCF-1 in turn binds with several transcription factors, resulting in the inhibition of cell proliferation. A mutated BAP-1 protein is unable to interact with HCF-1; as a result, the inhibitory effects of HCF-1 on cell proliferation are lost ^26^.

### The mTOR pathway

**Figure 2.**
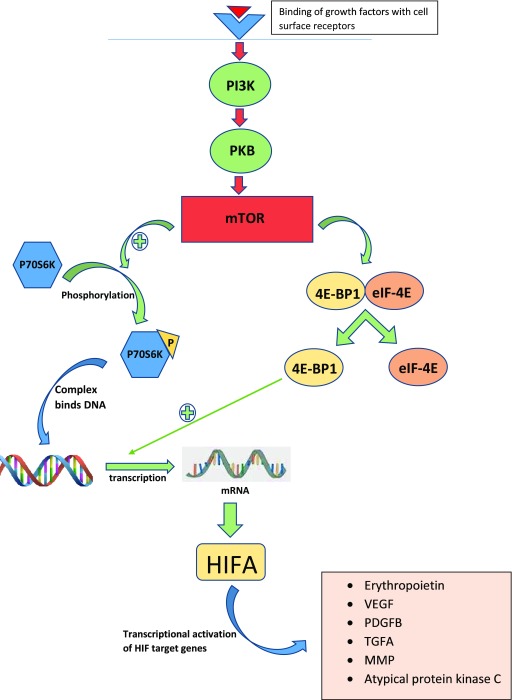
The mTOR–PI3K pathway. Binding of cell surface growth factors activates phosphatidylinositol-3-kinase (PI3K) protein, which in turn activates mTOR, creating mTOR complexes 1 and 2 (mTORC1 and mTORC2), resulting in the phosphorylation of P70S6K and 4E-BP1/eIF4E and increased production of angiogenic proteins such as VEGF, PDGF, and TGFB, leading to cell growth and tumor progression. 4E-BP1, 4E-binding protein 1; eIF-4E, eukaryotic initiation factor-4E; HIF, hypoxia-inducible factor; HIFA, hypoxia-inducible factor alpha; MMP, matrix metalloproteinase; mRNA, messenger RNA; mTOR, mammalian target of rapamycin; PDGFB, platelet-derived growth factor beta; PI3K, phosphatidylinositol-3-kinase; PKB, protein kinase B; TGFA, transforming growth factor alpha; VEGF, vascular endothelial growth factor.

The mammalian target of rapamycin (mTOR) is a protein kinase that is encoded by the *MTOR* gene. It plays an important role in the regulation of the cell cycle and has been a therapeutic target of interest in many other cancers as well. The mTOR–PI3K pathway starts with the binding of several growth factors to the cell surface, resulting in the activation of phosphatidylinositol-3-kinase (PI3K) protein ( Figure 2). Activated PI3K in turn activates mTOR, creating mTOR complexes 1 and 2 (mTORC1 and mTORC2), which lead to the phosphorylation of P70S6K and 4E-BP1/eukaryotic translation initiation factor 4E (4E-BP1/eIF4E). The phosphorylated P70SK migrates to the nucleus and initiates the transcription of mRNA coding for the HIFA protein, which, as mentioned above, has the ability to increase the production of angiogenic proteins such as VEGF, PDGF, and TGFB ^27– 29^. Phosphorylation of the translational regulator eIF4E-binding protein 1 (4E-BP1) also mediates the effects of oncogenic Akt signaling on mRNA translation, cell growth, and tumor progression ^29^.

Everolimus and temsirolimus are mTOR inhibitors that are approved for the treatment of metastatic ccRCC ^30, 31^. Additionally, the combination of lenvatinib (VEGFR inhibitor) and everolimus (mTOR inhibitor) has been approved for the treatment of metastatic RCC in the second- and subsequent-line setting.

## The role of the immune system in renal cell carcinoma

The cytotoxic component of the immune system plays a vital role in the recognition and subsequent rejection of several different types of cancer, including RCC. Unfortunately, this innate system is not always adequate in attacking and eliminating cancer. In order to survive the immune response, the cancer cells develop certain proteins on their cell surface, which help them fight off the cytotoxic T cells by certain pathways. One such pathway is the programmed death-1 (PD-1) pathway.

Under normal circumstances, the cytotoxic immune system is designed to recognize the “foreign antigens” present on the surface of cancer cells. This should lead to the activation of cytotoxic T lymphocytes, resulting in the release of cytokines such as interferons, interleukin-2, and tumor necrosis factor. These cytokines are directly responsible for the death of cancer cells. From a therapeutic standpoint, both interferon-alpha and interleukin-2 have historically been used for the treatment of ccRCC, although their use has diminished with the advent of effective and better-tolerated alternatives ^32, 33^. Immune T-cell infiltration is a prevalent characteristic of ccRCCs and represents an important target for immune checkpoint inhibitor therapy ^1, 34^.

### The programmed death-1 receptor pathway

**Figure 3.**
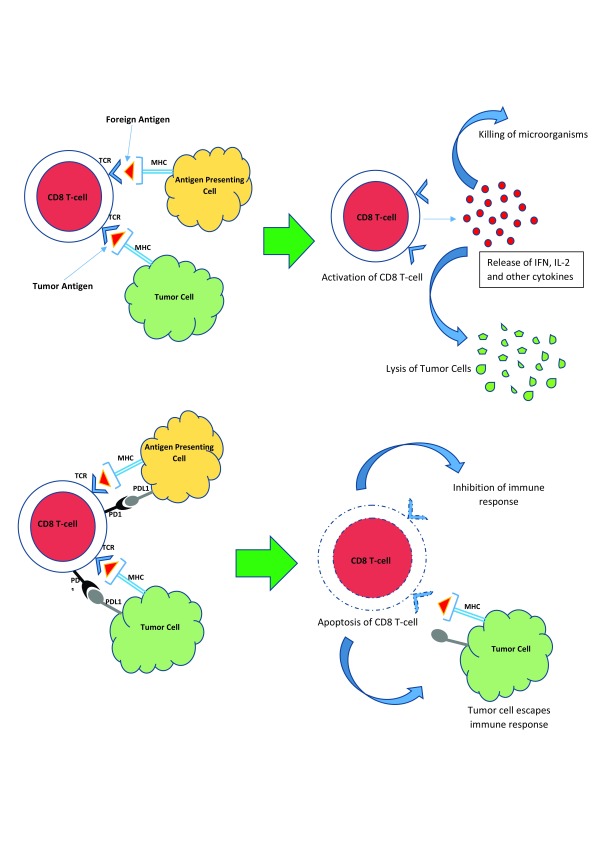
The programmed death-1 receptor pathway. Recognition of foreign or tumor antigens normally results in the activation of CD8 T cells, leading to the killing of viruses or bacteria or the lysis of tumor cells. The programmed death-1 (PD-1) receptor is a cell membrane protein present on the surface of CD8 T cells, which, upon interacting with PD-L1 and PD-L2 on the surface of tumor cells, results in the suppression of the cytotoxic immune response, leading to tumor escape.IFN, interferon; IL-2, interleukin-2; MHC, major histocompatibility complex; PD-1, programmed death-1; PD-L1, programmed death ligand-1; TCR, T-cell receptor.

The PD-1 receptor is a cell membrane protein present on the surface of cytotoxic T lymphocytes (CD8 T cells). The proteins that activate PD-1 are also cell surface proteins called programmed death ligand-1 and -2 (PD-L1 and PD-L2). PD-L1 is present on the surface of antigen-presenting cells and certain malignant cells, including RCC cells. The major function of PD-1, after being activated by binding PD-L1, is to suppress the cytotoxic immune system by inducing apoptosis of the cytotoxic T lymphocyte. This normally prevents an uncontrolled and unchecked autoimmune response when the body is exposed to a foreign antigen (such as a virus or bacterium). Thus, PD-1 is an “anti-immune” protein, the stimulation of which suppresses the immune system and decreases the number of cytotoxic T cells attacking foreign antigens and cancer cells ( Figure 3).

Several cancers, including ccRCC, express PD-L1 on their surface. The expression of PD-L1 allows these cancers to escape the cytotoxic immune response by inducing the apoptosis of cytotoxic T lymphocytes. This discovery has led to the development of antibodies that target and block PD-1 and PD-L1. The PD-1-blocking antibodies currently available are nivolumab and pembrolizumab. Nivolumab is currently approved for the treatment of metastatic ccRCC in the second-line setting ^35, 36^. Atezolizumab and avelumab are PD-L1-blocking monoclonal antibodies that are also under investigation for metastatic ccRCC.

CTLA-4 is a protein receptor present on the surface of cytotoxic T lymphocytes, and the primary function is downregulation of the immune system (similar to the PD-1/PD-L1 pathway) but by a different mechanism. An inhibitor of this receptor, called ipilimumab, in combination with PD-1/PD-L1 inhibitors, is under investigation and has shown promising results for the treatment of metastatic RCC ^37^.

OX-40 is another “pro-inflammatory” protein present on the surface of cytotoxic T lymphocytes. The stimulation of this receptor results in the activation of the immune system (similar to that seen with PD-1/PD-L1 inhibition). OX-40 agonists in combination with PD-1 inhibitors are under investigation for the treatment of RCC ^38^.

## Metabolic pathways and targets in renal cell carcinoma

Altered metabolic pathways in cancer cells play a key role in their survival. These pathways also help cancer cells escape different levels of stress imposed on them by the immune system as well as various drugs. Targeting various metabolic pathways in RCC is an active area of research in oncology ^39, 40^.

### The glutaminase pathway

Glutamine is an important amino acid in many cancer cells (including ccRCC cells) and is indirectly required by these cells for the synthesis of DNA. Cancer cells metabolize glutamine differently than do normal cells and require an enzyme called glutaminase. This enzyme converts glutamine to glutamate, which in turn increases the production of aspartate through the Krebs cycle. Aspartate plays a key role in the synthesis of pyrimidines in these cells and is required for maturation. In *VHL*-mutated/-deficient cells, glutamine is also required for the synthesis of essential lipids, citrate, and glutathione, the latter of which is the key anti-oxidant molecule generated by cells to combat oxidative stress ^41^. Therefore, inhibition of this pathway results in both cell cycle arrest (by decreasing pyrimidine synthesis required for DNA formation) and an inability of these cells to fight oxidative stress. These findings have led to the development of drugs targeting this pathway, which are under investigation. For example, CB-839 is a glutaminase inhibitor that is being studied in clinical trials for several solid tumors, including ccRCC ^42, 43^.

### Other metabolic pathways and targets

Glucose metabolism in cancer cells is also an area of clinical interest in this field. GLUT-1 is a glucose transporter that is responsible for the uptake of glucose by RCC cells. Several drugs blocking this transporter have been shown to be active in RCC ^44, 45^. Studies have demonstrated that, unlike in normal cells, glycolysis is the major source of glucose in cancer cells, even in the presence of sufficient oxygen (aerobic glycolysis) ^46^. As a result, several inhibitors of glycolytic enzymes have been investigated because of their activity in cancer cells, including RCC cells ^47, 48^. Similarly, pyruvate dehydrogenase inhibitors have shown activity in preclinical RCC xenografts ^49, 50^.

### Other novel approaches and combinations

Despite our extensive knowledge and understanding of the biology of this cancer, metastatic RCC continues to lead to a high number of deaths in the modern era. With the discovery of pathways outlined in this article, many research studies are now geared toward combining drugs that target different pathways in ccRCC. Combination studies in metastatic ccRCC that pair VEGF inhibition and checkpoint inhibition include bevacizumab plus atezolizumab, axitinib plus pembrolizumab, and nivolumab plus ipilimumab plus cabozantinib. There are also studies of combination immunotherapies targeting PD-1 and PD-L1 or targeting PD-L1 and OX40.

In addition, the glutaminase inhibitor CB-839 is being investigated in combination with anti-PD-1 antibody nivolumab as well as with the mTOR inhibitor everolimus and the VEGFR/MET inhibitor cabozantinib in separate clinical trials.

A better understanding of the biology and pathogenesis of ccRCC has revolutionized the treatment approach. Novel targets and combination strategies are under investigation with the hope of achieving longer and more durable responses and improving survival.

In summary, with a greater understanding of the pathogenesis of RCC, many therapeutic advances for metastatic disease have been introduced in the last decade. Such progress has led to improved survival for patients with advanced RCC. Much of the recent therapeutic development has focused on inhibition of the VEGFR and the mTOR pathways. Current and ongoing areas of research include the additional development of new immunotherapy targets, metabolic targeting, and combinational approaches to treat the disease.

## References

[1] Hsieh JJ Purdue MP Signoretti S: Renal cell carcinoma. *Nat Rev Dis Primers.* 2017;3:17009. 10.1038/nrdp.2017.9 28276433 PMC5936048

[2] Chow WH Dong LM Devesa SS: Epidemiology and risk factors for kidney cancer. *Nat Rev Urol.* 2010;7(5):245–57. 10.1038/nrurol.2010.46 20448658 PMC3012455

[3] Siegel RL Miller KD Jemal A: Cancer Statistics, 2017. *CA Cancer J Clin.* 2017;67(1):7–30. 10.3322/caac.21387 28055103

[4] Thompson RH Ordonez MA Iasonos A: Renal cell carcinoma in young and old patients--is there a difference? *J Urol.* 2008;180(4):1262–6; discussion 1266. 10.1016/j.juro.2008.06.037 18707708 PMC2615196

[5] Siegel R Ward E Brawley O: Cancer statistics, 2011: the impact of eliminating socioeconomic and racial disparities on premature cancer deaths. *CA Cancer J Clin.* 2011;61(4):212–36. 10.3322/caac.20121 21685461

[6] Siegel R Naishadham D Jemal A: Cancer statistics, 2012. *CA Cancer J Clin.* 2012;62(1):10–29. 10.3322/caac.20138 22237781

[7] Motzer RJ Jonasch E Agarwal N: Kidney Cancer, Version 2.2017, NCCN Clinical Practice Guidelines in Oncology. *J Natl Compr Canc Netw.* 2017;15(6):804–34. 10.6004/jnccn.2017.0100 28596261

[8] Beroukhim R Brunet JP Di Napoli A: Patterns of gene expression and copy-number alterations in von-hippel lindau disease-associated and sporadic clear cell carcinoma of the kidney. *Cancer Res.* 2009;69(11):4674–81. 10.1158/0008-5472.CAN-09-0146 19470766 PMC2745239

[9] Foster K Prowse A van den Berg A: Somatic mutations of the von Hippel-Lindau disease tumour suppressor gene in non-familial clear cell renal carcinoma. *Hum Mol Genet.* 1994;3(12):2169–73. 10.1093/hmg/3.12.2169 7881415

[10] Gnarra JR Tory K Weng Y: Mutations of the VHL tumour suppressor gene in renal carcinoma. *Nat Genet.* 1994;7(1):85–90. 10.1038/ng0594-85 7915601

[11] Yao M Yoshida M Kishida T: VHL tumor suppressor gene alterations associated with good prognosis in sporadic clear-cell renal carcinoma. *J Natl Cancer Inst.* 2002;94(20):1569–75. 10.1093/jnci/94.20.1569 12381710

[12] Nickerson ML Jaeger E Shi Y: Improved identification of von Hippel-Lindau gene alterations in clear cell renal tumors. *Clin Cancer Res.* 2008;14(15):4726–34. 10.1158/1078-0432.CCR-07-4921 18676741 PMC2629664

[13] Kaelin WG Jr: Molecular basis of the VHL hereditary cancer syndrome. *Nat Rev Cancer.* 2002;2(9):673–82. 10.1038/nrc885 12209156

[14] Barry RE Krek W: The von Hippel-Lindau tumour suppressor: a multi-faceted inhibitor of tumourigenesis. *Trends Mol Med.* 2004;10(9):466–72. 10.1016/j.molmed.2004.07.008 15350900

[15] Kim WY Kaelin WG: Role of *VHL* gene mutation in human cancer. *J Clin Oncol.* 2004;22(24):4991–5004. 10.1200/JCO.2004.05.061 15611513

[16] Pugh CW Ratcliffe PJ: Regulation of angiogenesis by hypoxia: role of the HIF system. *Nat Med.* 2003;9(6):677–84. 10.1038/nm0603-677 12778166

[17] Maxwell PH Pugh CW Ratcliffe PJ: Activation of the HIF pathway in cancer. *Curr Opin Genet Dev.* 2001;11(3):293–9. 10.1016/S0959-437X(00)00193-3 11377966

[18] Motzer RJ Hutson TE Tomczak P: Sunitinib versus interferon alfa in metastatic renal-cell carcinoma. *N Engl J Med.* 2007;356(2):115–24. 10.1056/NEJMoa065044 17215529

[19] Motzer RJ Hutson TE Cella D: Pazopanib versus sunitinib in metastatic renal-cell carcinoma. *N Engl J Med.* 2013;369(8):722–31. 10.1056/NEJMoa1303989 23964934

[20] Chen W Hill H Christie A: Targeting renal cell carcinoma with a HIF-2 antagonist. *Nature.* 2016;539(7627):112–7. 10.1038/nature19796 27595394 PMC5340502

[21] Cho H Du X Rizzi JP: On-target efficacy of a HIF-2α antagonist in preclinical kidney cancer models. *Nature.* 2016;539(7627):107–11. 10.1038/nature19795 27595393 PMC5499381

[22] Courtney KD Infante JR Lam ET: Phase I Dose-Escalation Trial of PT2385, a First-in-Class Hypoxia-Inducible Factor-2α Antagonist in Patients With Previously Treated Advanced Clear Cell Renal Cell Carcinoma. *J Clin Oncol.* 2017;JCO2017742627. 10.1200/JCO.2017.74.2627 29257710 PMC5946714

[23] Thompson M: Polybromo-1: the chromatin targeting subunit of the PBAF complex. *Biochimie.* 2009;91(3):309–19. 10.1016/j.biochi.2008.10.019 19084573 PMC2646799

[24] Brugarolas J: PBRM1 and BAP1 as novel targets for renal cell carcinoma. *Cancer J.* 2013;19(4):324–32. 10.1097/PPO.0b013e3182a102d1 23867514 PMC4222578

[25] Kapur P Peña-Llopis S Christie A: Effects on survival of *BAP1* and *PBRM1* mutations in sporadic clear-cell renal-cell carcinoma: a retrospective analysis with independent validation. *Lancet Oncol.* 2013;14(2):159–67. 10.1016/S1470-2045(12)70584-3 23333114 PMC4674067

[26] Peña-Llopis S Vega-Rubín-de-Celis S Liao A: BAP1 loss defines a new class of renal cell carcinoma. *Nat Genet.* 2012;44(7):751–9. 10.1038/ng.2323 22683710 PMC3788680

[27] Zoncu R Efeyan A Sabatini DM: mTOR: from growth signal integration to cancer, diabetes and ageing. *Nat Rev Mol Cell Biol.* 2011;12(1):21–35. 10.1038/nrm3025 21157483 PMC3390257

[28] Zarogoulidis P Lampaki S Turner JF: mTOR pathway: A current, up-to-date mini-review (Review). *Oncol Lett.* 2014;8(6):2367–70. 10.3892/ol.2014.2608 25360163 PMC4214394

[29] Laplante M Sabatini DM: mTOR signaling in growth control and disease. *Cell.* 2012;149(2):274–93. 10.1016/j.cell.2012.03.017 22500797 PMC3331679

[30] Motzer RJ Escudier B Oudard S: Efficacy of everolimus in advanced renal cell carcinoma: a double-blind, randomised, placebo-controlled phase III trial. *Lancet.* 2008;372(9637):449–56. 10.1016/S0140-6736(08)61039-9 18653228

[31] Rini BI Bellmunt J Clancy J: Randomized phase III trial of temsirolimus and bevacizumab versus interferon alfa and bevacizumab in metastatic renal cell carcinoma: INTORACT trial. *J Clin Oncol.* 2014;32(8):752–9. 10.1200/JCO.2013.50.5305 24297945

[32] Klapper JA Downey SG Smith FO: High-dose interleukin-2 for the treatment of metastatic renal cell carcinoma : a retrospective analysis of response and survival in patients treated in the surgery branch at the National Cancer Institute between 1986 and 2006. *Cancer.* 2008;113(2):293–301. 10.1002/cncr.23552 18457330 PMC3486432

[33] Fyfe G Fisher RI Rosenberg SA: Results of treatment of 255 patients with metastatic renal cell carcinoma who received high-dose recombinant interleukin-2 therapy. *J Clin Oncol.* 1995;13(3):688–96. 10.1200/JCO.1995.13.3.688 7884429

[34] Şenbabaoğlu Y Gejman RS Winer AG: Tumor immune microenvironment characterization in clear cell renal cell carcinoma identifies prognostic and immunotherapeutically relevant messenger RNA signatures. *Genome Biol.* 2016;17(1):231. 10.1186/s13059-016-1092-z 27855702 PMC5114739

[35] Motzer RJ Rini BI McDermott DF: Nivolumab for Metastatic Renal Cell Carcinoma: Results of a Randomized Phase II Trial. *J Clin Oncol.* 2015;33(13):1430–7. 10.1200/JCO.2014.59.0703 25452452 PMC4806782

[36] McDermott DF Drake CG Sznol M: Survival, Durable Response, and Long-Term Safety in Patients With Previously Treated Advanced Renal Cell Carcinoma Receiving Nivolumab. *J Clin Oncol.* 2015;33(18):2013–20. 10.1200/JCO.2014.58.1041 25800770 PMC4517051

[37] Hammers HJ Plimack ER Infante JR: Safety and Efficacy of Nivolumab in Combination With Ipilimumab in Metastatic Renal Cell Carcinoma: The CheckMate 016 Study. *J Clin Oncol.* 2017;35(34):3851–8. 10.1200/JCO.2016.72.1985 28678668 PMC7587408

[38] Linch SN McNamara MJ Redmond WL: OX40 Agonists and Combination Immunotherapy: Putting the Pedal to the Metal. *Front Oncol.* 2015;5:34. 10.3389/fonc.2015.00034 25763356 PMC4329814

[39] Pinthus JH Whelan KF Gallino D: Metabolic features of clear-cell renal cell carcinoma: mechanisms and clinical implications. *Can Urol Assoc J.* 2011;5(4):274–82. 21801687 10.5489/cuaj.10196 PMC3147044

[40] van der Mijn JC Panka DJ Geissler AK: Novel drugs that target the metabolic reprogramming in renal cell cancer. *Cancer Metab.* 2016;4:14. 10.1186/s40170-016-0154-8 27418963 PMC4944519

[41] Okazaki A Gameiro PA Christodoulou D: Glutaminase and poly(ADP-ribose) polymerase inhibitors suppress pyrimidine synthesis and *VHL*-deficient renal cancers. *J Clin Invest.* 2017;127(5):1631–45. 10.1172/JCI87800 28346230 PMC5409089

[42] Abu Aboud O Habib SL Trott J: Glutamine Addiction in Kidney Cancer Suppresses Oxidative Stress and Can Be Exploited for Real-Time Imaging. *Cancer Res.* 2017;77(23):6746–58. 10.1158/0008-5472.CAN-17-0930 29021138 PMC5791889

[43] Meric-Bernstam F Tannir NM Mier JW: Phase 1 study of CB-839, a small molecule inhibitor of glutaminase (GLS), alone and in combination with everolimus (E) in patients (pts) with renal cell cancer (RCC). *J Clin Oncol.* 2016;34(15_suppl):4568 Reference Source

[44] Chan DA Sutphin PD Nguyen P: Targeting GLUT1 and the Warburg effect in renal cell carcinoma by chemical synthetic lethality. *Sci Transl Med.* 2011;3(94):94ra70. 10.1126/scitranslmed.3002394 21813754 PMC3683134

[45] Liu Y Cao Y Zhang W: A small-molecule inhibitor of glucose transporter 1 downregulates glycolysis, induces cell-cycle arrest, and inhibits cancer cell growth *in vitro* and *in vivo*. *Mol Cancer Ther.* 2012;11(8):1672–82. 10.1158/1535-7163.MCT-12-0131 22689530

[46] WARBURG O: On respiratory impairment in cancer cells. *Science.* 1956;124(3215):269–70. 13351639

[47] Clem B Telang S Clem A: Small-molecule inhibition of 6-phosphofructo-2-kinase activity suppresses glycolytic flux and tumor growth. *Mol Cancer Ther.* 2008;7(1):110–20. 10.1158/1535-7163.MCT-07-0482 18202014

[48] Clem BF O'Neal J Tapolsky G: Targeting 6-phosphofructo-2-kinase (PFKFB3) as a therapeutic strategy against cancer. *Mol Cancer Ther.* 2013;12(8):1461–70. 10.1158/1535-7163.MCT-13-0097 23674815 PMC3742633

[49] Papandreou I Cairns RA Fontana L: HIF-1 mediates adaptation to hypoxia by actively downregulating mitochondrial oxygen consumption. *Cell Metab.* 2006;3(3):187–97. 10.1016/j.cmet.2006.01.012 16517406

[50] Kinnaird A Dromparis P Saleme B: Metabolic Modulation of Clear-cell Renal Cell Carcinoma with Dichloroacetate, an Inhibitor of Pyruvate Dehydrogenase Kinase. *Eur Urol.* 2016;69(4):734–44. 10.1016/j.eururo.2015.09.014 26433571

